# 3,3′-Dimethyl-1,1′-methyl­ene­diimidazolium dibromide

**DOI:** 10.1107/S1600536809028967

**Published:** 2009-07-29

**Authors:** Jie Liu, Xilian Wei, Zengbin Wei, Jiuqiang Liu, Liqiang Zheng

**Affiliations:** a Key Laboratory of Colloid and Interface Chemistry, Shandong University, Jinan 250100, People’s Republic of China; b College of Chemistry and Chemical Engineering, Liaocheng University, Liaocheng 252059, People’s Republic of China

## Abstract

In the crystal structure of the title compound, C_9_H_14_N_4_^2+^·2Br^−^, the cation and anions have crystallographic mirror symmetry, with the mirror plane running through the central CH_2_ group for the cation. The latter are stacked along the *a* axis, forming channels hosting the bromide anions. The crystal packing is stabilized by C—H⋯Br hydrogen-bonding inter­actions, generating a two-dimensional network.

## Related literature

For related structures, see: Jin *et al.* (2007 ▶); Eicher *et al.* (2003 ▶).
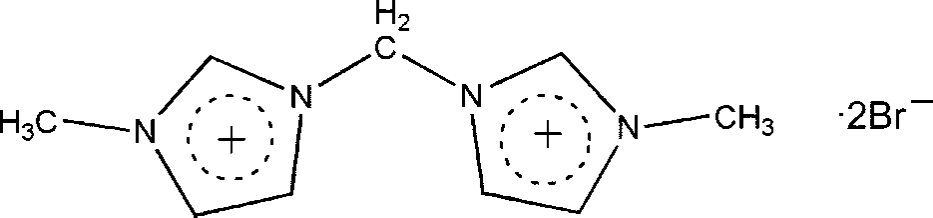


## Experimental

### 

#### Crystal data

C_9_H_14_N_4_^2+^·2Br^−^
*M* *_r_* = 338.06
Monoclinic, 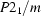

*a* = 4.7310 (5) Å
*b* = 11.3861 (12) Å
*c* = 11.8419 (15) Å
β = 93.672 (1)°
*V* = 636.59 (12) Å^3^
*Z* = 2
Mo *K*α radiation
μ = 6.34 mm^−1^
*T* = 298 K
0.32 × 0.10 × 0.07 mm

#### Data collection

Bruker SMART diffractometer
Absorption correction: multi-scan (*SADABS*; Sheldrick, 1996 ▶) *T*_min_ = 0.227, *T*_max_ = 0.638
3349 measured reflections
1188 independent reflections
928 reflections with *I* > 2σ(*I*)
*R*_int_ = 0.031

#### Refinement

*R*[*F* ^2^ > 2σ(*F* ^2^)] = 0.028
*wR*(*F* ^2^) = 0.066
*S* = 1.07
1188 reflections
74 parameters
H-atom parameters constrained
Δρ_max_ = 0.63 e Å^−3^
Δρ_min_ = −0.27 e Å^−3^

### 

Data collection: *SMART* (Siemens, 1996 ▶); cell refinement: *SAINT* (Siemens, 1996 ▶); data reduction: *SAINT*; program(s) used to solve structure: *SHELXS97* (Sheldrick, 2008 ▶); program(s) used to refine structure: *SHELXL97* (Sheldrick, 2008 ▶); molecular graphics: *SHELXTL* (Sheldrick, 2008 ▶); software used to prepare material for publication: *SHELXTL*.

## Supplementary Material

Crystal structure: contains datablocks I, global. DOI: 10.1107/S1600536809028967/rz2350sup1.cif

Structure factors: contains datablocks I. DOI: 10.1107/S1600536809028967/rz2350Isup2.hkl

Additional supplementary materials:  crystallographic information; 3D view; checkCIF report

## Figures and Tables

**Table 1:** Hydrogen-bond geometry (Å, °)

*D*—H⋯*A*	*D*—H	H⋯*A*	*D*⋯*A*	*D*—H⋯*A*
C4—H4⋯Br1^i^	0.93	2.84	3.724 (3)	158
C3—H3⋯Br1^ii^	0.93	2.81	3.699 (3)	160
C1—H1*A*⋯Br1^iii^	0.97	2.76	3.723 (4)	172
C2—H2⋯Br2^iv^	0.93	2.82	3.627 (3)	146
C1—H1*B*⋯Br2^iv^	0.97	2.81	3.652 (5)	146

Symmetry codes: (i) 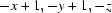; (ii) 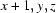; (iii) 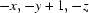; (iv) 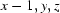.

**Fig. 1.**
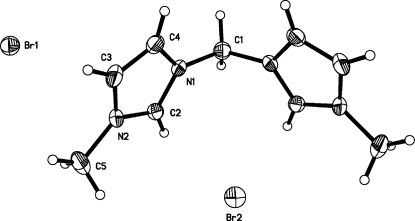
The molecular structure of the compound, with atom labels and 50% probability displacement ellipsoids. Unlabelled atoms are related to labelled atoms by (x, 0.5-y, z)

**Fig. 2.**
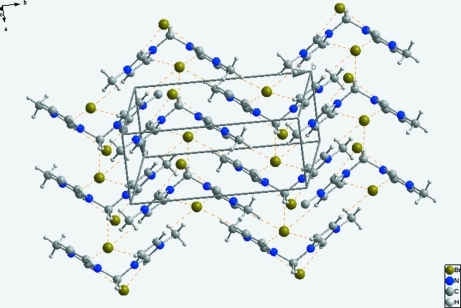
Crystal packing of the compound, showing the two-dimensional network structure formed by C—H···Br hydrogen bonds (dashed lines).

## supplementary crystallographic information

### Comment

The title compound was synthesized as the precursor of a chelating N-heterocyclic carbene ligand, which can be generated by deprotonating the ring between the two N atoms of the two imidazolium cations.(Jin *et al.*, 2007).

The structure consists of dimethylethylenediimidazolium cations and bromide anions (Fig. 1). The cation has crystallographically imposed mirror symmetry, with atom C1 located on a mirror plane. Both independent bromide anions also lie on a mirror plane. The C1—N1 bond length is 1.455 (4) Å, and the N1—C1—N1 bond angle is 111.0 (4)°. The C2—N1—C4 bond angle of 108.4 (3)° is similar to those observed in free imidazole (Eicher *et al.*, 2003). The relative orientation of the imidazolium ring with respect to the other imidazolium ring can be described by the value of -95.4 (4)° of the C2—N1—C1—N1 torsion angle. In the crystal, the cations are stacked along the *a* axis forming channels that are occupied by the bromide anions (Fig. 2). Adjacent molecules are connected into a two-dimensional network through C—H···Br hydrogen interactions (Table 1).

### Experimental

A mixture of 1-methylimidazole (0.1 mol) and dichloromethane (0.05 mol) was reacted under nitrogen atmosphere with stirring at 350 K for 48 h. The resulting clear solution was evaporated under vacuum. Colourless crystals suitable for X-ray analysis were obtained by slow evaporation of a ethyl acetate solution over a period of two weeks. (yield 83%) Anal. Calcd (%) for C_9_H_14_Br_2_N_4_ (Mr = 338.06): C, 32.03; H, 4.09; N, 16.62. Found (%): C, 31.95; H, 4.14; N, 16.57.

### Refinement

All H atoms were placed geometrically and treated as riding on their parent atoms, with C—H = 0.93–0.97 Å, and with *U*_iso_(H) = 1.2*U*_eq_(C) or 1.5*U*_eq_(C) for methyl H atoms.

### Crystal data

**Table:**

C_9_H_14_N_4_^2+^·2Br^−^	*F*(000) = 332
*M**_r_* = 338.06	*D*_x_ = 1.764 Mg m^−^^3^
Monoclinic, *P*2_1_/*m*	Mo *K*α radiation, λ = 0.71073 Å
Hall symbol: -P 2yb	Cell parameters from 1657 reflections
*a* = 4.7310 (5) Å	θ = 2.5–26.3°
*b* = 11.3861 (12) Å	µ = 6.34 mm^−^^1^
*c* = 11.8419 (15) Å	*T* = 298 K
β = 93.672 (1)°	Block, colourless
*V* = 636.59 (12) Å^3^	0.32 × 0.10 × 0.07 mm
*Z* = 2	

### Data collection

**Table:**

Bruker SMART diffractometer	1188 independent reflections
Radiation source: fine-focus sealed tube	928 reflections with *I* > 2σ(*I*)
graphite	*R*_int_ = 0.031
φ and ω scans	θ_max_ = 25.0°, θ_min_ = 1.7°
Absorption correction: multi-scan (*SADABS*; Sheldrick, 1996)	*h* = −5→5
*T*_min_ = 0.227, *T*_max_ = 0.638	*k* = −13→10
3349 measured reflections	*l* = −14→13

### Refinement

**Table:**

Refinement on *F*^2^	Primary atom site location: structure-invariant direct methods
Least-squares matrix: full	Secondary atom site location: difference Fourier map
*R*[*F*^2^ > 2σ(*F*^2^)] = 0.028	Hydrogen site location: inferred from neighbouring sites
*wR*(*F*^2^) = 0.066	H-atom parameters constrained
*S* = 1.07	*w* = 1/[σ^2^(*F*_o_^2^) + (0.0319*P*)^2^] where *P* = (*F*_o_^2^ + 2*F*_c_^2^)/3
1188 reflections	(Δ/σ)_max_ < 0.001
74 parameters	Δρ_max_ = 0.63 e Å^−^^3^
0 restraints	Δρ_min_ = −0.27 e Å^−^^3^

### Fractional atomic coordinates and isotropic or equivalent isotropic displacement parameters (Å^2^)

**Table:**

	*x*	*y*	*z*	*U*_iso_*/*U*_eq_	
Br1	0.14906 (11)	0.7500	0.12842 (4)	0.04708 (18)	
Br2	0.86811 (10)	0.2500	0.43062 (4)	0.04621 (18)	
N1	0.3731 (5)	0.3553 (2)	0.18410 (19)	0.0333 (6)	
N2	0.5612 (5)	0.5023 (2)	0.2736 (2)	0.0375 (6)	
C1	0.2048 (10)	0.2500	0.1615 (4)	0.0410 (11)	
H1A	0.1319	0.2500	0.0831	0.049*	
H1B	0.0447	0.2500	0.2088	0.049*	
C2	0.3825 (7)	0.4151 (3)	0.2809 (2)	0.0361 (8)	
H2	0.2796	0.3978	0.3431	0.043*	
C3	0.6745 (7)	0.4977 (3)	0.1702 (3)	0.0450 (8)	
H3	0.8081	0.5490	0.1436	0.054*	
C4	0.5580 (7)	0.4061 (3)	0.1145 (3)	0.0421 (8)	
H4	0.5953	0.3814	0.0421	0.050*	
C5	0.6396 (9)	0.5872 (3)	0.3625 (3)	0.0699 (13)	
H5A	0.4742	0.6296	0.3820	0.105*	
H5B	0.7764	0.6412	0.3360	0.105*	
H5C	0.7197	0.5466	0.4280	0.105*	

### Atomic displacement parameters (Å^2^)

**Table:**

	*U*^11^	*U*^22^	*U*^33^	*U*^12^	*U*^13^	*U*^23^
Br1	0.0526 (4)	0.0461 (3)	0.0433 (3)	0.000	0.0087 (2)	0.000
Br2	0.0433 (3)	0.0484 (3)	0.0471 (3)	0.000	0.0040 (2)	0.000
N1	0.0389 (16)	0.0264 (14)	0.0339 (14)	0.0034 (12)	−0.0035 (12)	0.0049 (12)
N2	0.0483 (17)	0.0230 (14)	0.0405 (15)	−0.0001 (14)	−0.0022 (13)	−0.0011 (12)
C1	0.041 (3)	0.037 (3)	0.043 (3)	0.000	−0.008 (2)	0.000
C2	0.043 (2)	0.0321 (18)	0.0327 (16)	0.0052 (16)	0.0014 (14)	−0.0011 (14)
C3	0.053 (2)	0.0311 (18)	0.051 (2)	−0.0003 (17)	0.0103 (17)	0.0093 (17)
C4	0.055 (2)	0.0358 (18)	0.0355 (17)	0.0082 (17)	0.0063 (16)	0.0059 (16)
C5	0.109 (4)	0.042 (2)	0.058 (3)	−0.019 (2)	0.003 (2)	−0.0142 (19)

### Geometric parameters (Å, °)

**Table:**

N1—C2	1.331 (3)	C1—H1B	0.9700
N1—C4	1.368 (4)	C2—H2	0.9300
N1—C1	1.455 (4)	C3—C4	1.334 (4)
N2—C2	1.311 (4)	C3—H3	0.9300
N2—C3	1.370 (4)	C4—H4	0.9300
N2—C5	1.459 (4)	C5—H5A	0.9600
C1—N1^i^	1.455 (4)	C5—H5B	0.9600
C1—H1A	0.9700	C5—H5C	0.9600
			
C2—N1—C4	108.4 (3)	N1—C2—H2	125.8
C2—N1—C1	124.6 (3)	C4—C3—N2	107.4 (3)
C4—N1—C1	127.0 (3)	C4—C3—H3	126.3
C2—N2—C3	108.7 (3)	N2—C3—H3	126.3
C2—N2—C5	126.1 (3)	C3—C4—N1	107.0 (3)
C3—N2—C5	125.1 (3)	C3—C4—H4	126.5
N1—C1—N1^i^	111.0 (4)	N1—C4—H4	126.5
N1—C1—H1A	109.4	N2—C5—H5A	109.5
N1^i^—C1—H1A	109.4	N2—C5—H5B	109.5
N1—C1—H1B	109.4	H5A—C5—H5B	109.5
N1^i^—C1—H1B	109.4	N2—C5—H5C	109.5
H1A—C1—H1B	108.0	H5A—C5—H5C	109.5
N2—C2—N1	108.5 (3)	H5B—C5—H5C	109.5
N2—C2—H2	125.8		
			
C2—N1—C1—N1^i^	−95.4 (4)	C2—N2—C3—C4	0.6 (4)
C4—N1—C1—N1^i^	80.7 (4)	C5—N2—C3—C4	177.6 (3)
C3—N2—C2—N1	−1.1 (3)	N2—C3—C4—N1	0.2 (4)
C5—N2—C2—N1	−178.2 (3)	C2—N1—C4—C3	−0.9 (4)
C4—N1—C2—N2	1.2 (3)	C1—N1—C4—C3	−177.5 (3)
C1—N1—C2—N2	177.9 (3)		

Symmetry codes: (i) *x*, −*y*+1/2, *z*.

### Hydrogen-bond geometry (Å, °)

**Table:**

*D*—H···*A*	*D*—H	H···*A*	*D*···*A*	*D*—H···*A*
C4—H4···Br1^ii^	0.93	2.84	3.724 (3)	158
C3—H3···Br1^iii^	0.93	2.81	3.699 (3)	160
C1—H1A···Br1^iv^	0.97	2.76	3.723 (4)	172
C2—H2···Br2^v^	0.93	2.82	3.627 (3)	146
C1—H1B···Br2^v^	0.97	2.81	3.652 (5)	146

Symmetry codes: (ii) −*x*+1, −*y*+1, −*z*; (iii) *x*+1, *y*, *z*; (iv) −*x*, −*y*+1, −*z*; (v) *x*−1, *y*, *z*.
